# Normal gastrointestinal mucosa at biopsy and subsequent cancer risk: nationwide population-based, sibling-controlled cohort study

**DOI:** 10.1186/s12885-022-09992-5

**Published:** 2022-08-13

**Authors:** Jiangwei Sun, Fang Fang, Ola Olén, Mingyang Song, Jonas Halfvarson, Bjorn Roelstraete, Hamed Khalili, Jonas F. Ludvigsson

**Affiliations:** 1 Institute of Environmental Medicine, Karolinska Institutet, Stockholm, Sweden; 2 Department of Medicine Solna, Clinical Epidemiology Division, Karolinska Institutet, Stockholm, Sweden; 3 Sachs’ Children and Youth Hospital, Stockholm South General Hospital, Stockholm, Sweden; 4 Department of Clinical Science and Education Södersjukhuset, Karolinska Institutet, Stockholm, Sweden; 5 Departments of Epidemiology and Nutrition, Harvard T.H. Chan School of Public Health, Boston, MA USA; 6 Clinical and Translational Epidemiology Unit, Massachusetts General Hospital and Harvard Medical School, Boston, MA USA; 7 Division of Gastroenterology, Massachusetts General Hospital and Harvard Medical School, Boston, MA USA; 8 Department of Gastroenterology, Faculty of Medicine and Health, Örebro University, Örebro, Sweden; 9 Department of Medical Epidemiology and Biostatistics, Karolinska Institutet, 171 77 Stockholm, Sweden; 10 Broad Institute of MIT and Harvard, Cambridge, MA 02142 USA; 11 Department of Pediatrics, Örebro University Hospital, Örebro, Sweden; 12 Department of Medicine, Division of Digestive and Liver Disease, Columbia University Medical Center, New York, NY USA

**Keywords:** Gastrointestinal, Histology, Normal mucosa, Cancer, Cohort, Screening

## Abstract

**Background:**

While individuals with normal gastrointestinal (GI) mucosa on endoscopy have a lower risk of colorectal cancer, risks of other cancers remain unexplored.

**Methods:**

Through Sweden’s 28 pathology departments, we identified 415,092 individuals with a first GI biopsy with histologically normal mucosa during 1965–2016 and no prior cancer. These individuals were compared to 1,939,215 matched reference individuals from the general population. Follow-up began 6 months after biopsy, and incident cancer data were retrieved from the Swedish Cancer Register. Flexible parametric model was applied to estimate cumulative incidences and hazard ratios (HRs) for cancers. We also used full siblings (*n* = 441,534) as a secondary comparison group.

**Results:**

During a median follow-up of 10.9 years, 40,935 individuals with normal mucosa (incidence rate: 82.74 per 10,000 person-years) and 177,350 reference individuals (incidence rate: 75.26) developed cancer. Restricting the data to individuals where follow-up revealed no cancer in the first 6 months, we still observed an increased risk of any cancer in those with a histologically normal mucosa (average HR = 1.07; 95%CI = 1.06–1.09). Although the HR for any and specific cancers decreased shortly after biopsy, we observed a long-term excess risk of any cancer, with an HR of 1.08 (95%CI = 1.05–1.12) and a cumulative incidence difference of 0.93% (95%CI = 0.61%-1.25%) at 30 years after biopsy. An elevated risk of gastric cancer, lung cancer, and hematological malignancy (including lymphoproliferative malignancy) was also observed at 20 or 30 years since biopsy. Sibling analyses confirmed the above findings.

**Conclusion:**

Individuals with a histologically normal mucosa at biopsy and where follow-up revealed no cancer in the first 6 months, may still be at increased risk of cancer, although excess risks are small.

**Supplementary Information:**

The online version contains supplementary material available at 10.1186/s12885-022-09992-5.

## Introduction

Gastrointestinal (GI) disease is common [1, 2], and requires extensive resources not only for treatment but also for the work-up of symptoms. Millions of endoscopies are performed in the US each year [3]. The most frequent histologic finding on endoscopy is a normal mucosa [4]. Earlier research by our group has shown that individuals with normal *duodenal/jejunal* mucosa on biopsy, albeit with positive celiac disease serology [5], are at increased risk of a wide range of diseases, including chronic obstructive pulmonary disease (COPD) [6], amyotrophic lateral sclerosis [7], autism [8], and epilepsy [9].

While earlier research has shown that individuals with a normal colorectal mucosa are at a lower risk of colorectal cancer (CRC) [10, 11], little is known about the overall risk of cancer in individuals with a histologically normal GI mucosa. We are unaware of any studies assessing risks of other cancers than CRC in patients with a normal mucosa. Moreover, information on the temporal pattern of cancer risk in relation to follow-up time after a biopsy of normal mucosa is largely unexplored. Such information could also help in identifying periods of increased cancer risk in clinical practice. Furthermore, cancer as well as risk factors for cancer tend to cluster within families, and it is yet unknown whether any increase in cancer risk in patients requiring an endoscopy that reveals a histologically normal mucosa is fully explained by these familial factors.

We therefore undertook a comprehensive population-based, sibling-controlled cohort study in Sweden to explore the associations between a normal upper/lower GI biopsy and risk of cancer. We hypothesized that individuals with a normal upper/lower GI biopsy had a decreased risk for CRC but increased risk for other cancers. We compared these individuals with their individually matched population references as well as their unexposed full siblings to address the potential concern of confounding.

## Methods

### Study design and participants

Individuals with normal mucosa were retrieved from the nationwide histopathology cohort ESPRESSO (Epidemiology Strengthened by histoPathology Reports in Sweden) [12]. ESPRESSO was collected during 2015–2017 from all 28 pathology departments in Sweden, and included information from biopsy reports on topography (upper GI tract: T60-T65; and lower GI tract: T66-T69 or T6X) and morphology (through the Systematized Nomenclature of Medicine (SNOMED) system to code histopathology). We also requested data on the personal identity number assigned to all residents in Sweden [13], date of biopsy, as well as county of the pathology department.

Those with the first GI biopsy report of normal mucosa (SNOMED codes: M00100 and M00110) and without other aberration were identified as the exposed individuals. Hence, individuals with for instance an earlier record of celiac disease or inflammatory bowel disease (IBD), where subsequent biopsies may show a normal mucosa, were excluded. Our definition of normal mucosa has a high positive predictive value (> 98%) for both upper and lower GI biopsies with a result of normal mucosa [7].

For every exposed individual with a GI biopsy of normal mucosa, up to five reference individuals were randomly selected from the Swedish Total Population Register [14], which contains data on migrations, births, and deaths. The exposed and reference individuals were individually and exactly matched by birth year, sex, county of residence, and calendar period. The reference individuals had to be alive and biopsy-naïve at time of selection. We restricted our analysis to both exposed and unexposed individuals with no earlier record of cancer.

To reduce residual confounding from the shared genetics and early environmental factors within families [15], we also identified biopsy-naïve full siblings of the exposed individuals. The siblings had to be alive and had no prior GI biopsy on the date of biopsy of the exposed individual.

All study participants were then followed through cross-linkages to several national registers using the unique personal identity number [13]. End of follow-up was defined as date of incident cancer, a competing event (including death or emigration out of Sweden), or December 31, 2016, whichever came first. This cross-linkage guarantees a complete follow-up.

### Cancer ascertainment

The Swedish Cancer Register is estimated to cover > 97% of all cancer diagnoses in Sweden [16], and was used to identify new diagnoses of cancer during follow-up of the study cohort. All recorded cancers in Sweden are back-translated to the International Classification of Disease, version 7 (ICD-7) for the sake of consistency. The primary outcome was any newly diagnosed cancer (and first ever cancer of any type for that individual), and the secondary outcome was a diagnosis of specific cancers, including solid cancers (specifically GI, lung, and breast cancers) and hematological malignancies. We also studied gastric cancer, CRC, hepatobiliary cancer, pancreatic cancer, and lymphoproliferative malignancies. The latter was previously shown to be associated with various GI diseases [17–20]. The ICD-7 codes for different cancers can be found in eTable 1.

### Covariates

The following covariates were considered when examining cancer risk in individuals with a normal GI mucosa: (a). country of birth (Nordic vs. other country); (b). educational attainment (4 groups: 0–9 years, 10–12 years, ≥ 13 years, and “missing”; identified from the Swedish LISA database [21]); (c). number of healthcare visits between 2 years and 6 months prior to the biopsy (4 categories: 0, 1, 2–3, and ≥ 4), as a proxy for access to health care; (d). Charlson comorbidity index (3 categories: 0, 1, and ≥ 2; without considering ulcer disease), as a proxy for general health status [22]; and (e). history of GI disease prior to the biopsy (yes vs. no), as GI disease may result in endoscopy whereas mucosal healing is a management aim in many GI diseases [23].

### Statistical analysis

We used flexible parametric model to estimate the average hazard ratio (HR) as well as the temporal pattern of HR since biopsy, together with their 95% confidence intervals (CIs) [24]. Compared with Cox proportional hazard model, flexible parametric model allows the effect of a covariate to vary over time rather than being constant. We started follow-up from 6 months after biopsy to avoid detection bias at the time of biopsy, or as part of simultaneous cancer work-up (where biopsy may have been one of several investigations). Standardized cumulative incidence of cancer was also estimated for the exposed individuals and their matched references and siblings using the flexible parametric model [25].

In addition to reporting average HR, we further presented the HR and cumulative incidence at 1 year, 5 years, 10 years, 20 years, and 30 years after biopsy for each outcome, as the HR and cumulative incidence may vary with follow-up time. In the model, we conditioned on the matching variables (birth year, sex, county of residence, and calendar period), and further adjusted for country of birth, educational attainment, number of healthcare visits, Charlson comorbidity index, and history of GI disease.

To assess whether the associations would differ in different subgroups, we carried out a number of subgroup analyses for any cancer, including age at cohort entry (< 18 y, 18–39.9 y, 40–59.9 y, and ≥ 60 y), sex, biopsy location (upper vs. lower GI tract), start of follow-up (1969–1989, 1990–1999, 2000–2009, and 2010–2016), and number of healthcare visit (0, 1, 2–3, and ≥ 4). We stratified the analyses by biopsy location to estimate the potentially varied impact of normal mucosa on the risk of specific cancers.

### Sensitivity analyses

We performed a number of sensitivity analyses to account for potential influence from comorbidity and healthcare utilization. We restricted the analyses to: (a). individuals with a Charlson comorbidity index of zero; (b). individuals free of GI diseases before biopsy; (c). individuals without a record of endoscopy before biopsy (for relevant codes, see eTable 2); and (d). individuals without an earlier record of colectomy or proctocolectomy (for relevant codes, see eTable 2). Moreover, we performed sibling analyses to minimize residual confounding from shared genetic and early environmental factors as well as healthcare seeking behavior that may result in a GI biopsy within families. Finally, to assess the impact of choice of start of follow-up, we started follow-up from one year after time of biopsy/selection in the analysis for the risk of any cancer.

Statistical analyses were carried out using SAS version 9.4 (SAS Institute Inc, Cary, NC), Stata (version 15.0; StataCorp LP, College Station, TX), and R version 3.6.0 [26]. We considered a two-sided *P* ≤ 0.05 as statistically significant.

### Ethics

The present study was approved by the Ethical Review Board in Stockholm. Due to its register-based nature, individual informed consent was waived by the board [27].

## Results

We retrieved data from 415,092 individuals with a GI biopsy of histologically normal mucosa and no earlier record of cancer (Table 1). Of these, 61.0% were female and the mean age at cohort entry was 42.6 years. Most had undergone an upper endoscopy (60.7%); and about 2 out of 3 patients had been first biopsied after year 2000. Compared with their matched reference individuals (*n* = 1,939,215), individuals with a histologically normal GI mucosa at biopsy had more healthcare visits between 2 years and 6 months prior to biopsy date, as well as a higher frequency of an earlier record of comorbidity, GI disease, endoscopy, and colectomy or proctocolectomy. The median follow-up was 10.9 years among individuals with a normal mucosa, and 11.1 years among the reference individuals (Table 1).

**Table 1:** Characteristics of individuals with a gastrointestinal (GI) biopsy result of normal mucosa and their matched references, a nationwide matched cohort study in Sweden, 1965–2016

Characteristics	No. (%)	
	Normal mucosa (*n* = 415,092)	Reference (*n* = 1,939,215)
Age at cohort entry, years ^a^		
Mean ± SD	42.6 ± 19.9	41.4 ± 19.6
Median (IQR)	41.6 (26.5, 57.6)	40.2 (25.8, 55.9)
< 18 y	37,162 (9.0)	185,918 (9.6)
18–39.9 y	159,128 (38.3)	776,081 (40.0)
40–59.9 y	128,543 (31.0)	597,589 (30.8)
≥ 60 y	90,259 (21.7)	379,627 (19.6)
Sex		
Male	161,935 (39.0)	774,466 (39.9)
Female	253,157 (61.0)	1,164,749 (60.1)
Country of birth		
Nordic country	372,414 (89.7)	1,695,737 (87.4)
Other country	42,678 (10.3)	243,478 (12.6)
Biopsy location ^b^		
Upper GI	252,130 (60.7)	1,178,909 (60.8)
Lower GI	162,962 (39.3)	760,306 (39.2)
Calendar period of cohort entry		
1969–1989	37,065 (8.9)	175,750 (9.1)
1990–1999	105,966 (25.5)	497,625 (25.7)
2000–2009	159,364 (38.4)	741,892 (38.3)
2010–2016	112,697 (27.2)	523,948 (27.0)
Educational attainment		
0–9 y	87,205 (21.0)	401,459 (20.7)
10–12 y	155,033 (37.4)	705,301 (36.4)
≥ 13 y	103,714 (25.0)	486,016 (25.1)
Missing	69,140 (16.7)	346,439 (17.9)
Number of healthcare visit		
0	262,249 (63.2)	1,474,064 (76.0)
1	63,749 (15.4)	231,583 (11.9)
2–3	48,551 (11.7)	144,925 (7.5)
≥ 4	40,543 (9.8)	88,643 (4.6)
Charlson comorbidity index		
0	336,937 (81.2)	1,732,278 (89.3)
1	58,881 (14.2)	165,699 (8.5)
≥ 2	19,274 (4.6)	41,238 (2.1)
History before start of follow-up		
GI disease	192,744 (46.4)	269,048 (13.9)
Endoscopy	129,815 (31.3)	40,494 (2.1)
Colectomy or proctocolectomy	283 (0.1)	275 (0.0)
Follow-up years since biopsy		
Median (IQR)	10.9 (5.7, 17.8)	11.1 (5.8, 18.2)
0.5–1 y	5460 (1.3)	22,315 (1.2)
1–4.9 y	82,598 (19.9)	378,898 (19.5)
5–9.9 y	102,809 (24.8)	471,651 (24.3)
10–19.9 y	147,159 (35.5)	687,486 (35.5)
20–29.9 y	64,044 (15.4)	313,228 (16.2)
≥ 30 y	13,022 (3.1)	65,637 (3.4)

*IQR* Interquartile range, *SD* Standard deviation

^a^Cohort entry: date of first biopsy record for individuals with a gastrointestinal biopsy result of normal mucosa, and date of selection for the matched references

^b^References were assigned a value of biopsy location based on the index person

During follow-up (beginning 6 months after biopsy), 40,935 (incidence rate: 82.74 per 10,000 person-years) individuals with normal mucosa and 177,350 (incidence rate: 75.26 per 10,000 person-years) reference individuals developed cancer (median and interquartile range of time to cancer diagnosis: 8.2 (3.7, 14.2) years), with an incidence rate difference of 7.48 (95%CI: 6.61, 8.35) per 10,000 person-years (eTable 3). A slightly higher incidence rate for hepatobiliary cancer, pancreatic cancer, lung cancer, breast cancer (women), and hematological malignancy (including lymphoproliferative malignancy), but lower incidence rate for CRC, was seen for individuals with a GI biopsy of normal mucosa (eTable 3).

### General population comparison: any cancer

**Fig. 1:**
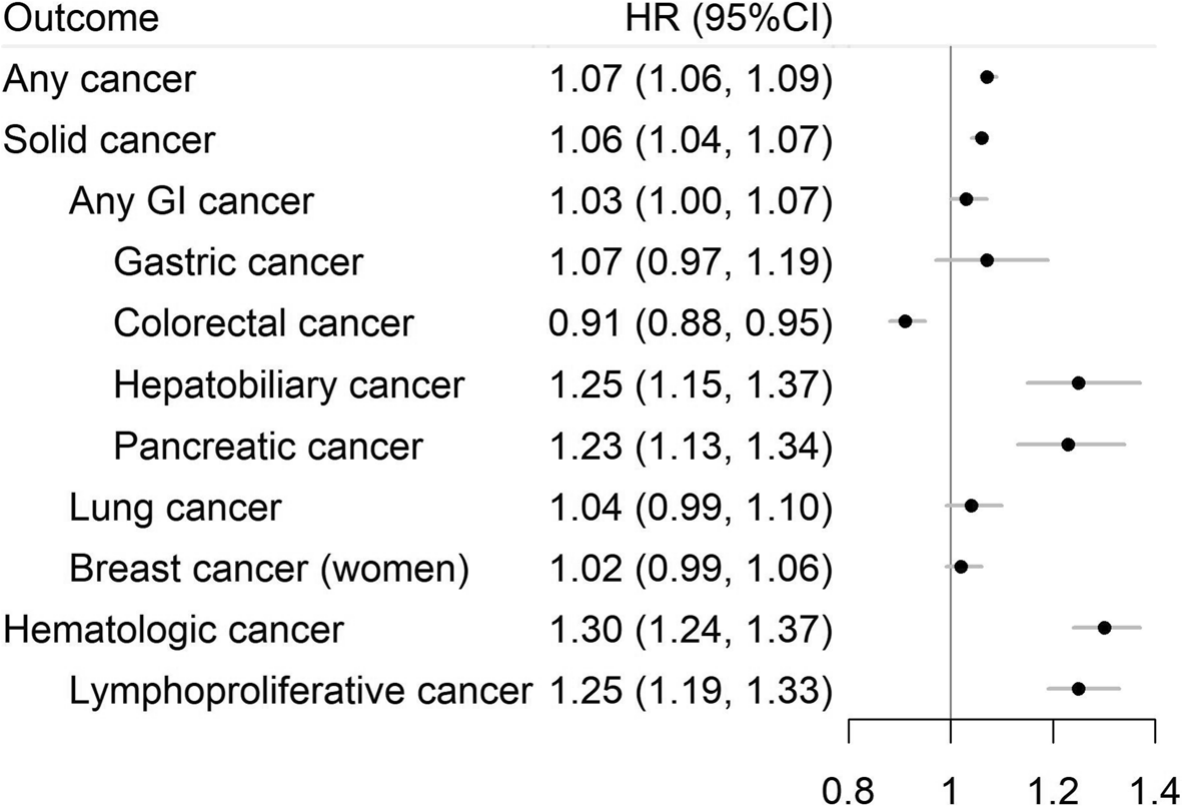
Average hazard ratio (HR) and 95% confidence intervals (CIs) of any and specific cancers, comparing individuals with a GI biopsy result of normal mucosa with their matched references, estimated from the flexible parametric model. Follow-up was started 6 months after the biopsy

**Fig. 2:**
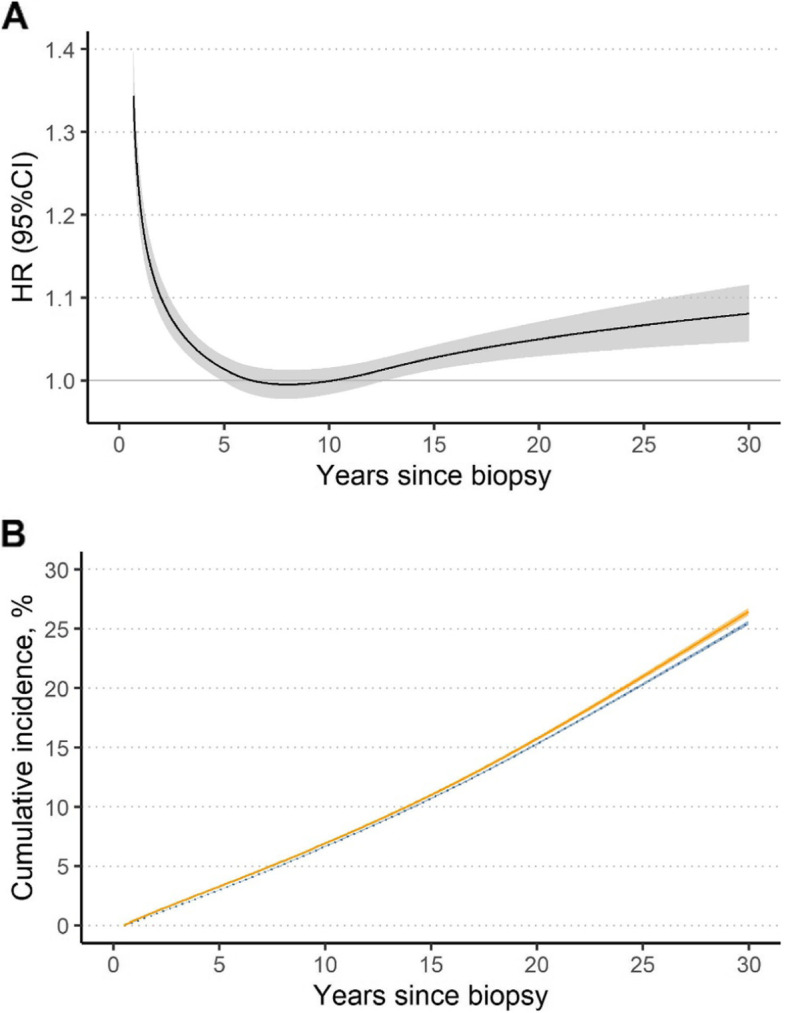
**A** Hazard ratio (HR) and 95% confidence intervals (CIs) of any cancer, comparing individuals with a GI biopsy result of normal mucosa with their matched references; **B** Standardized cumulative incidence and 95% confidence intervals of any cancer in individuals with normal mucosa (solid line and orange) and their matched references (dotted line and blue). Both were estimated from the flexible parametric model and follow-up was started 6 months after the biopsy

After excluding the first 6 months of follow-up from the analysis, we observed an increased risk of any cancer in those with a GI biopsy of normal mucosa (average HR = 1.07; 95%CI = 1.06–1.09, Fig. 1). The highest HRs were seen shortly after biopsy, which decreased rapidly towards 1 or even lower until around 13 years after biopsy (Fig. 2A). Thereafter, there was a consistently increased risk of any cancer until 30 years since biopsy. The HR for any cancer at 1 year after biopsy was 1.21 (95%CI = 1.18, 1.25), followed by HRs below or around one at 5 and 10 years, and > 1.0 again at 20 years (HR = 1.05; 95%CI = 1.03, 1.07) and 30 years (HR = 1.08; 95%CI = 1.05, 1.12) after biopsy (Table 2).

**Table 2:** Risk of any cancer during follow-up in individuals with a GI biopsy result of normal mucosa, compared with their matched references and siblings

	Years since biopsy				
	1 y	5 y	10 y	20 y	30 y
Compared with matched references ^a^					
HR (95%CIs)	1.21 (1.18, 1.25)	1.01 (1.00, 1.03)	1.00 (0.98, 1.02)	1.05 (1.03, 1.07)	1.08 (1.05, 1.12)
Cumulative incidence (95% CIs), %					
Reference	0.32 (0.31, 0.33)	3.00 (2.98, 3.03)	6.67 (6.63, 6.71)	15.33 (15.25, 15.42)	25.57 (25.40, 25.75)
Normal mucosa	0.44 (0.42, 0.45)	3.29 (3.24, 3.34)	6.93 (6.85, 7.01)	15.77 (15.62, 15.92)	26.50 (26.19, 26.82)
Difference	0.11 (0.10, 0.13)	0.29 (0.23, 0.34)	0.26 (0.17, 0.35)	0.43 (0.28, 0.59)	0.93 (0.61, 1.25)
Compared with their siblings ^b^					
HR (95%CIs)	1.40 (1.33, 1.48)	1.12 (1.09, 1.15)	1.05 (1.02, 1.08)	1.03 (1.00, 1.07)	1.03 (0.97, 1.08)
Cumulative incidence (95% CIs), %					
Siblings	0.20 (0.19, 0.21)	1.96 (1.92, 2.00)	4.67 (4.60, 4.74)	12.13 (11.97, 12.30)	22.11 (21.75, 22.49)
Normal mucosa	0.30 (0.28, 0.32)	2.41 (2.36, 2.47)	5.30 (5.20, 5.39)	12.92 (12.72, 13.13)	23.00 (22.55, 23.46)
Difference	0.10 (0.08, 0.12)	0.46 (0.39, 0.53)	0.63 (0.51, 0.74)	0.79 (0.58, 1.00)	0.89 (0.42, 1.36)

*CIs* Confidence intervals, *GI* Gastrointestinal, *HR* Hazard ratio

^a^Conditioned on matching set (birth year, sex, county of residence, and calendar period) and further adjusted for country of birth, educational attainment, number of healthcare visits, Charlson comorbidity index, and history of GI diseases

^b^Conditioned on family identifiers and further adjusted for birth year, sex, county of residence, calendar period, country of birth, educational attainment, number of healthcare visits, Charlson comorbidity index, and history of GI diseases

The cumulative incidence for any cancer was constantly higher among individuals undergoing a biopsy with normal mucosa compared with reference individuals (Fig. 2B). For example, the cumulative incidence for any cancer at 1 year, 10 years and 30 years after biopsy was 0.44%, 6.93%, and 26.50% in the exposed individuals compared with 0.32%, 6.67%, and 25.57% among reference individuals (Table 2). This translated into small differences in cumulative incidence, namely at 1 year 0.11% (95%CI = 0.10%, 0.13%), 10 years 0.26% (95%CI = 0.17%, 0.35%), and 30 years 0.93% (95%CI = 0.61%, 1.25%) after biopsy (Table 2).

In subgroup analyses, a stronger association of normal mucosa with any cancer was observed in males (HR = 1.11; 95%CI = 1.09, 1.13) than females (HR = 1.07; 95%CI = 1.05, 1.08), and for upper GI biopsy (HR = 1.08; 95%CI = 1.07, 1.10) than lower GI biopsy (HR = 1.05; 95%CI = 1.03, 1.07) (eTable 4). Higher HRs, but lower cumulative incidences, of any cancer were observed in younger adults and in the earliest and latest calendar periods (eTable 4, eFigure 1, and eFigure 2). Similar results were observed in different strata of number of healthcare visit (eTable 4).

### General population comparison: specific cancers

**Fig. 3:**
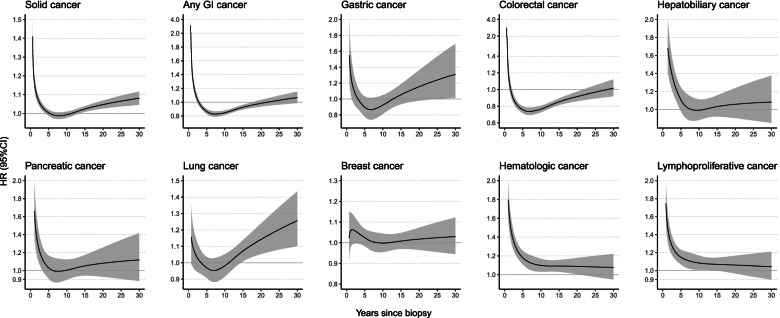
Hazard ratio (HR) and 95% confidence intervals (CIs) of specific cancers, comparing individuals with a GI biopsy result of normal mucosa with their matched references, estimated from the flexible parametric model. Follow-up was started 6 months after the biopsy

We observed an elevated risk of almost all specific cancers (HRs ranged from 1.02 to 1.30), except for CRC (HR = 0.91; 95%CI = 0.88, 0.95) (Fig. 1). Although different cancers have varying temporal pattern of HR, the increased HRs were mainly observed shortly after biopsy (Fig. 3). The HR decreased to 1 more quickly for GI cancers than non-GI cancers (e.g., CRC: 1.6 years; gastric cancer: 3 years; and hematological malignancies: > 30y). The highest HR at 1 year after biopsy was seen for hepatobiliary cancer (HR = 1.87; 95%CI = 1.54, 2.27) (eTable 5). At 1 year, we also noted an around 1.7-fold increased risk of pancreatic cancer and hematologic malignancy, specifically lymphoproliferative malignancy (HR = 1.64; 95%CI = 1.44, 1.86). Thirty years after biopsy, we could still detect excess risks for gastric cancer (HR = 1.31; 95%CI = 1.01, 1.69) and lung cancer (HR = 1.26; 95%CI = 1.10, 1.44), but not for lymphoproliferative malignancies (HR = 1.04; 95%CI = 0.90, 1.21) (eTable 5). eTable 6 and eFigure 3 show the cancer-specific cumulative incidence and its difference between individuals with normal mucosa and their reference individuals.

In subgroup analyses by biopsy location, stronger associations tend to be found for upper GI biopsy (eFigure 4). For example, the HR of any GI cancer was 1.10 (95%CI = 1.06, 1.14) among those with upper GI biopsy of normal mucosa, but not among those with lower GI biopsy with normal mucosa (HR = 0.90; 95%CI: 0.85, 0.95), which may be mainly driven by the decreased risk of CRC (HR = 0.76; 95%CI: 0.71, 0.82).

### Sensitivity analyses

Results were similar when we restricted the analysis to individuals with a Charlson comorbidity index of zero, free of GI diseases, no earlier record of endoscopy, or no prior colectomy or proctocolectomy before cohort entry (eTable 7). All sensitivity analyses yielded HRs between 1.19 and 1.21 at 1 year after biopsy, and at 20 years after biopsy, those with a GI biopsy of normal mucosa were again at a minimally increased risk of any cancer (HRs ranged from 1.05 to 1.06) (eTable 7).

### Sibling comparison

Individuals with a histologically normal GI mucosa and their full siblings (*n* = 441,534) had similar age (36.7 vs. 37.7 years), but individuals with normal mucosa were more often female (60.8% vs. 47.9%) than their siblings (eTable 8). Besides, individuals with a GI biopsy of normal mucosa still had a higher number of healthcare visits and higher frequency of GI disease and comorbidity.

Compared with their full siblings, individuals with a GI biopsy of normal mucosa were at an increased risk of any cancer (Table 2). The HR for any cancer at 1 year, 10 years and 30 years after biopsy was 1.40 (95%CI = 1.33, 1.48), 1.05 (95%CI = 1.02, 1.08), and 1.03 (95%CI = 0.97, 1.08), respectively, with a cumulative incidence difference of 0.89% (95%CI = 0.42%, 1.36%) at 30 years. The temporal pattern of HR and cumulative incidence was similar to that of the population comparison (eFigure 5). After excluding the first year after time of biopsy/selection from the analysis, the average HR for any cancer attenuated slightly, but the HRs at 5 years, 10 years, 20 years, and 30 years after biopsy were similar (eTable 9).

## Discussion

Adults with a histologically normal GI mucosa are generally regarded as low-risk individuals for CRC [28–31], but data on non-CRC cancers are scarce. In this population-based and sibling-controlled cohort study, we found a distinct pattern of cancer risk among individuals with a GI biopsy of normal mucosa. After excluding the first 6 months after biopsy, we observed an increased risk of any cancer and almost all specific cancers, except for CRC. Although the increased risk was mainly observed shortly after biopsy (particularly for GI cancers), there was a consistently increased risk of any cancer from around 13 years and onwards. An elevated risk of gastric cancer, lung cancer and hematological malignancy (including lymphoproliferative malignancy) was also observed at 20 or 30 years since biopsy. The increased risk was consistently observed when comparing these people with their full siblings.

Most individuals in our study probably had endoscopy with biopsy due to GI symptoms or deviating laboratory test results, some of which were likely attributable to an underlying cancer, often detected in the first 6 months after biopsy. While asymptomatic screening for CRC started in the Swedish Stockholm-Gotland region in 2008 (fecal occult blood test or fecal immunochemical test in people aged 60–69 years), this is unlikely to have affected our results more than marginally given the limited age span and that only 1 in 5 Swedes live in this part of the country. Hence, very few individuals in our study had their biopsy as part of general CRC screening. National screening endoscopy was only recently introduced in Sweden. Still, if only a minority of lower GI endoscopies were due to screening, this may explain the slightly lower cancer risk among individuals with a normal lower GI mucosa compared to those with a normal upper GI mucosa.

The high HR for any or specific cancers shortly after the biopsy mirrors earlier studies where for instance normal duodenal/jejunal mucosa in the presence of celiac disease antibodies has been associated with very high risks of GI cancer just after biopsy, but not beyond the first year of follow-up [32]. Similarly, we have previously shown high risks for CRC and small intestinal cancer shortly after IBD diagnosis, followed by substantially lower risk estimates with increasing follow-up [33–35], suggesting an impact of concomitant IBD-CRC or that an underlying cancer may have contributed to the diagnosis of the non-malignant disease (IBD) in the first place.

Of note, requiring a GI biopsy where the biopsy showed normal mucosa was also linked to an increased risk of lymphoproliferative malignancy (HR = 1.64 at 1 year after biopsy). Excess risks of lymphoproliferative malignancy have been demonstrated in a number of GI inflammatory disorders [36], although admittedly the cited Pedersen et al.study failed to attain statistical significance (standardized incidence ratio = 1.42; 95%CI = 0.95–2.12) [36]. The increased risk of lymphoproliferative malignancy has often been explained through the presence of inflammation [37], supported by the fact that in celiac disease, mucosal healing seems protective against future lymphoproliferative malignancy [38]. However, in the present study, individuals with a histologically normal GI mucosa were more prone to develop lymphoproliferative malignancy, both when compared with reference individuals and when compared with siblings. The increased cancer risk might not be due to the normal mucosa per se, rather that the presence of such a biopsy is a marker for GI symptoms or abnormal laboratory test results leading to an endoscopy. Incomplete endoscopies and missed cancers at time of first biopsy with normal mucosa might explain the rapid initial risk increase after biopsy. This would underline that a normal GI mucosa in patients with symptoms suggestive of cancer does not rule out cancer in the near future. The excess risk vanished around 5 years after biopsy, which might be a consequence of the depletion of susceptible individuals due to the premature diagnosis of cancer in the first several years. Moreover, residual confounding might also contribute to the increased cancer risk. For instance, individuals undergoing an endoscopy may be different from the general population whereas smokers and heavy alcohol consumers are probably more likely to undergo cancer-related investigations than others.

While it should be emphasized that albeit the overall HRs decreased shortly after biopsy, we did observe a small long-term excess risk of any cancer, with an HR of 1.08 (95%CI = 1.05–1.12) and a cumulative incidence difference of 0.93% (95%CI = 0.61%, 1.25%) at 30 years after biopsy. After 20 or 30 years of follow-up, the excess cancer-specific risks were also seen for gastric cancer, lung cancer, and hematological malignancy (including lymphoproliferative malignancy). Of note much of the increase was driven by biopsies obtained before 1990s during a period when access to advanced radiology may have been poorer than today (i.e., cancers might to a greater extent not have been detected within 6 months of the biopsy). This might suggest that these HRs and cumulative incidences may not be fully applicable to individuals with a GI biopsy of normal mucosa today. Regardless, as HRs varied over time periods of follow-up, differences in follow-up periods have also contributed to the fact that people with different calendar periods of cohort entry had different average HRs.

### Strengths and limitations

Strengths include the nationwide population-based and sibling-controlled cohort design with a large sample size (> 400,000 individuals with a biopsy of normal mucosa vs. around 2 million references from the general population) and long and virtually complete follow-up due to its register-based nature. It enables us to present the HR and cumulative incidence up to 30 years after biopsy and to carry out a number of subgroup and sensitivity analyses, examining for example men and women separately as well as noting somewhat stronger associations in younger compared with elderly people. Objective and prospective ascertainment of normal mucosa and cancers minimized the potential information bias often seen in observational studies. An earlier validation study has shown that > 98% of individuals with a record of normal mucosa had a consistent biopsy report [7]. Using two different measures (HRs and cumulative incidence over time), we could explore the association in terms of both relative and absolute risks. Moreover, sibling comparisons allayed concern about the potential influence of familial confounding, further adding to our extensive adjustment for potential confounders. Consistent and robust findings across multiple subgroup and sensitivity analyses provide high-quality evidence for the cancer risk among individuals with a histologically normal mucosa but no cancer diagnosis during the first 6 months after biopsy. This might have important clinical implications. For example, clinicians should be aware of the potentially increased risk of future cancer, either relatively short term (beyond six months after biopsy and mainly for GI cancers) or long term (e.g., for gastric, lung, and hematologic cancers), among these individuals and inform patients that they should seek for healthcare service again if other alarming symptoms appear.

There are also some limitations. We acknowledge that there may exist residual confounding from lifestyle factors such as physical exercise, obesity, and smoking. All these factors may influence future cancer risk and an individual’s chance to undergo endoscopy. While such concern may be partly relieved by the similar results between the population and sibling comparisons, as these factors tend to cluster within families, we cannot rule out that e.g., more prevalent smoking in individuals undergoing an endoscopy with normal mucosa may have contributed to the long-term increased risk of lung cancer and gastric cancer. Second, we lacked data on indications for biopsy and therefore cannot rule out that some individuals that had an undiagnosed GI disease at time of biopsy and some individuals that underwent a normal endoscopy but never had a mucosal biopsy were included in the reference individuals. Third, the individuals with a biopsy of normal mucosa had a higher average Charlson comorbidity index and more healthcare visits before biopsy, potentially contributing to greater risk of cancer diagnosis. This is however not likely a major concern in present study as we adjusted for Charlson comorbidity index and number of healthcare visits in all analyses and found largely unchanged results in the sensitivity analyses after restricting the analysis to individuals with a Charlson comorbidity index of zero or individuals without healthcare visits between 2 years and 6 months prior to cohort entry. Finally, we had no data on screening (e.g., fecal occult blood test or fecal immunochemical test) or on endoscopic quality or macroscopic appearance.

## Conclusion

In conclusion, individuals with GI symptoms requiring an endoscopy, where the GI biopsy was histologically normal, were still at an increased risk of cancer 6 months or more after their endoscopy. They tended to have an increased long-term risk of gastric cancer, lung cancer, and hematological malignancy, but consistent with earlier research, a lower risk of CRC.

## Supplementary Information

**Additional file 1: eTable 1. **ICD-7 codes for cancer outcomes. **eTable 2. **Definitions of endoscopy, colectomy, and proctocolectomy. **eTable 3.** Incidence rate of cancer in individuals with a GI biopsy result of normal mucosa and their matched population references. **eTable 4.** Average risk of any cancer during follow-up in individuals with a GI biopsy result of normal mucosa and their matched references. **eTable 5.** Risk of specific cancers during follow-up in individuals with a GI biopsy result of normal mucosa and their matched references. **eTable 6.** Cumulative incidence and its difference of specific cancers during follow-up in individuals with a GI biopsy result of normal mucosa and their matched references. **eTable 7.** Sensitivity analyses for the risk of any cancer during follow-up in individuals with a GI biopsy result of normal mucosa and their matched references. **eTable 8.** Characteristics of individuals with a gastrointestinal (GI) biopsy result of normal mucosa and their siblings, a nationwide matched cohort study in Sweden, 1965-2016. **eTable 9.** Association between a GI biopsy result of normal mucosa and risk of any cancer, starting follow-up from 6 months vs. 1 year after biopsy. **eFigure 1.** Standardized cumulative incidence and 95% confidence intervals of any cancer in individuals with a GI biopsy result of normal mucosa (solid line and orange) and their matched references (dotted line and blue), stratified by age at cohort entry or calendar period of cohort entry. Follow-up was started 6 months after the biopsy. **eFigure 2.** Hazard ratio (HR) and 95% confidence intervals (CIs) of any cancer as a function of time since biopsy, comparing individuals with a GI biopsy result of normal mucosa with their matched references, stratified by calendar period of cohort entry. **eFigure 3.** Standardized cumulative incidence and 95% confidence intervals of specific cancers in individuals with a GI biopsy result of normal mucosa (solid line and orange) and their matched references (dotted line and blue). Follow-up was started 6 months after the biopsy. **eFigure 4.** Average hazard ratio (HR) and 95% confidence intervals (CIs) of specific cancers, comparing individuals with a GI biopsy result of normal mucosa with their matched references, stratified by biopsy location: upper (blue) or lower (orange) GI. Follow-up was started 6 months after the biopsy. **eFigure 5.** (A). Hazard ratio (HR) and 95% confidence intervals (CIs) of any cancer, comparing individuals with a GI biopsy result of normal mucosa with their siblings; (B). Standardized cumulative incidence and 95% confidence intervals of any cancer in individuals with normal mucosa (solid line and orange) and their siblings (dotted line and blue). Both were estimated from the flexible parametric model and follow-up was started 6 months after the biopsy.

## Data Availability

The data that support the findings of this study are available from the Swedish National Board of Health, Statistics Sweden and Swedish pathology departments, but restrictions apply to the availability of these data, which were used under license for the current study, and so are not publicly available.

## Abbreviations

CI: Confidence interval
CRC: Colorectal cancer
ESPRESSO: Epidemiology Strengthened by histoPathology Reports in Sweden
GI: Gastrointestinal
HR: Hazard ratio
IBD: Inflammatory bowel disease
ICD: International Classification of Diseases
SNOMED: Systematized Nomenclature of Medicine
