# Supplementary material for: Normal gastrointestinal mucosa at biopsy and subsequent cancer risk: nationwide population-based, sibling-controlled cohort study
Source: BMC Cancer. 2022 Aug 13;22:890. doi: 10.1186/s12885-022-09992-5 (PMC9375922; doi:10.1186/s12885-022-09992-5)
Supplement: Supplementary file 1 — Additional file 1: eTable 1. ICD-7 codes for cancer outcomes. eTable 2. Definitions of endoscopy, colectomy, and proctocolectomy. eTable 3. Incidence rate of cancer in individuals with a GI biopsy result of normal mucosa and their matched population references. eTable 4. Average risk of any cancer during follow-up in individuals with a GI biopsy result of normal mucosa and their matched references. eTable 5. Risk of specific cancers during follow-up in individuals with a GI biopsy result of normal mucosa and their matched references. eTable 6. Cumulative incidence and its difference of specific cancers during follow-up in individuals with a GI biopsy result of normal mucosa and their matched references. eTable 7. Sensitivity analyses for the risk of any cancer during follow-up in individuals with a GI biopsy result of normal mucosa and their matched references. eTable 8. Characteristics of individuals with a gastrointestinal (GI) biopsy result of normal mucosa and their siblings, a nationwide matched cohort study in Sweden, 1965-2016. eTable 9. Association between a GI biopsy result of normal mucosa and risk of any cancer, starting follow-up from 6 months vs. 1 year after biopsy. eFigure 1. Standardized cumulative incidence and 95% confidence intervals of any cancer in individuals with a GI biopsy result of normal mucosa (solid line and orange) and their matched references (dotted line and blue), stratified by age at cohort entry or calendar period of cohort entry. Follow-up was started 6 months after the biopsy. eFigure 2. Hazard ratio (HR) and 95% confidence intervals (CIs) of any cancer as a function of time since biopsy, comparing individuals with a GI biopsy result of normal mucosa with their matched references, stratified by calendar period of cohort entry. eFigure 3. Standardized cumulative incidence and 95% confidence intervals of specific cancers in individuals with a GI biopsy result of normal mucosa (solid line and orange) and their matched references (do [file 12885_2022_9992_MOESM1_ESM.docx]

**Supplementary Materials**

**Normal gastrointestinal mucosa at biopsy and subsequent cancer risk: Nationwide population-based, sibling-controlled cohort study**

Sun J et al.

**Contents**

**Tables**

**eTable 1.** ICD-7 codes for cancer outcomes.

**eTable 2.** Definitions of endoscopy, colectomy, and proctocolectomy.

**eTable 3.** Incidence rate of cancer in individuals with a GI biopsy result of normal mucosa and their matched population references

**eTable 4.** Average risk of any cancer during follow-up in individuals with a GI biopsy result of normal mucosa and their matched references.

**eTable 5.** Risk of specific cancers during follow-up in individuals with a GI biopsy result of normal mucosa and their matched references.

**eTable 6.** Cumulative incidence and its difference of specific cancers during follow-up in individuals with a GI biopsy result of normal mucosa and their matched references.

**eTable 7.** Sensitivity analyses for the risk of any cancer during follow-up in individuals with a GI biopsy result of normal mucosa and their matched references.

**eTable 8.** Characteristics of individuals with a gastrointestinal (GI) biopsy result of normal mucosa and their siblings, a nationwide matched cohort study in Sweden, 1965-2016.

**eTable 9.** Association between a GI biopsy result of normal mucosa and risk of any cancer, starting follow-up from 6 months vs. 1 year after biopsy.

**Figures**

**eFigure 1.** Standardized cumulative incidence and 95% confidence intervals of any cancer in individuals with a GI biopsy result of normal mucosa (solid line and orange) and their matched references (dotted line and blue), stratified by age at cohort entry or calendar period of cohort entry. Follow-up was started 6 months after the biopsy.

**eFigure 2.** Hazard ratio (HR) and 95% confidence intervals (CIs) of any cancer as a function of time since biopsy, comparing individuals with a GI biopsy result of normal mucosa with their matched references, stratified by calendar period of cohort entry.

**eFigure 3.** Standardized cumulative incidence and 95% confidence intervals of specific cancers in individuals with a GI biopsy result of normal mucosa (solid line and orange) and their matched references (dotted line and blue). Follow-up was started 6 months after the biopsy.

**eFigure 4.** Average hazard ratio (HR) and 95% confidence intervals (CIs) of specific cancers, comparing individuals with a GI biopsy result of normal mucosa with their matched references, stratified by biopsy location: upper (blue) or lower (orange) GI. Follow-up was started 6 months after the biopsy.

**eFigure 5.** (A). Hazard ratio (HR) and 95% confidence intervals (CIs) of any cancer, comparing individuals with a GI biopsy result of normal mucosa with their siblings; (B). Standardized cumulative incidence and 95% confidence intervals of any cancer in individuals with normal mucosa (solid line and orange) and their siblings (dotted line and blue). Both were estimated from the flexible parametric model and follow-up was started 6 months after the biopsy.

| eTable 1. ICD-7 codes for cancer outcomes | |
| --- | --- |
| Outcome | ICD-7 code |
| Any cancer | 140-208 |
| Solid cancer | 140-199 |
| Gastrointestinal cancer | 150-159 |
| Gastric cancer | 151 |
| Colorectal cancer | 153-154 |
| Hepatobiliary cancer | 155-156 |
| Pancreatic cancer | 157 |
| Lung cancer | 162 |
| Breast cancer (women) | 170 |
| Hematologic cancer | 200-208 |
| Lymphoproliferative cancer | 200-204 |
| In the Cancer Register, all ICD-8, ICD-9, and ICD-10 codes were back-translated to ICD-7. | |

| eTable 2. Definitions of endoscopy, colectomy, and proctocolectomy | | |
| --- | --- | --- |
|  |  |  |
| Endoscopy | Esophagogastroduodenoscopy | 2861, 2880, 2881, 4480, 4483, 4486, 4487, 4488, 4489, 4490, 9021, 4686, 4687, 9003, 9004, 9021, UJC, UJD, UJF02, UJF05 |
|  | Colonoscopy or sigmoidoscopy | 9011, 9012, 9023, 4685, 4688, 4689, 4674, 4684, UJF32, UJF35, UJF42, UJF45 |
| Colectomy | Sixth revision | 4650, 4651 |
|  | Seventh revision | JFH10, JFH11, JFH96, JFH00, JFH01, JFC40, JFC41, JFG29, JFG26 |
| Proctocolectomy | Sixth revision | 4652, 4653, 4654 |
|  | Seventh revision | JFH30, JFH33, JGB50, JGB60, JFH40, JFH20 |

| eTable 3. Incidence rate of cancer in individuals with a GI biopsy result of normal mucosa and their matched population references | | | | | | | |
| --- | --- | --- | --- | --- | --- | --- | --- |
|  | No. of events, n (%) | |  | Incidence rate (95% CIs), per 10,000 Pys | |  | Incidence rate difference (95% CIs), per 10,000 Pys |
| Outcomes | Normal mucosa | References |  | Normal mucosa | References |  |  |
| Any cancer | 40935 (9.9) | 177350 (9.2) |  | 82.74 (81.94, 83.54) | 75.26 (74.91, 75.61) |  | 7.48 (6.61, 8.35) |
| Solid cancer | 38170 (9.2) | 166842 (8.6) |  | 77.15 (76.38, 77.93) | 70.80 (70.46, 71.14) |  | 6.35 (5.51, 7.19) |
| Any GI cancer | 6400 (1.5) | 29826 (1.5) |  | 12.94 (12.62, 13.26) | 12.66 (12.51, 12.80) |  | 0.28 (-0.07, 0.63) |
| Gastric cancer | 593 (0.1) | 2641 (0.1) |  | 1.20 (1.10, 1.30) | 1.12 (1.08, 1.16) |  | 0.08 (-0.03, 0.18) |
| Colorectal cancer | 3627 (0.9) | 19263 (1.0) |  | 7.33 (7.09, 7.57) | 8.17 (8.06, 8.29) |  | -0.84 (-1.11, -0.58) |
| Hepatobiliary cancer | 797 (0.2) | 2862 (0.2) |  | 1.61 (1.50, 1.73) | 1.21 (1.17, 1.26) |  | 0.40 (0.28, 0.52) |
| Pancreatic cancer | 786 (0.2) | 3107 (0.2) |  | 1.59 (1.48, 1.70) | 1.32 (1.27, 1.37) |  | 0.27 (0.15, 0.39) |
| Lung cancer | 2224 (0.5) | 9543 (0.5) |  | 4.50 (4.31, 4.69) | 4.05 (3.97, 4.13) |  | 0.45 (0.24, 0.65) |
| Breast cancer (women) | 5559 (2.2) | 24720 (2.1) |  | 18.22 (17.74, 18.70) | 17.33 (17.11, 17.54) |  | 0.89 (0.37, 1.42) |
| Hematologic cancer | 2765 (0.7) | 10508 (0.5) |  | 5.59 (5.38, 5.80) | 4.46 (4.37, 4.55) |  | 1.13 (0.90, 1.35) |
| Lymphoproliferative cancer | 1999 (0.5) | 7877 (0.4) |  | 4.04 (3.87, 4.22) | 3.34 (3.27, 3.42) |  | 0.70 (0.51, 0.89) |

| eTable 4. Average risk of any cancer during follow-up in individuals with a GI biopsy result of normal mucosa and their matched references | |
| --- | --- |
|  | Average HR (95% CI) |
| Sex |  |
| Male | 1.11 (1.09, 1.13) |
| Female | 1.07 (1.05, 1.08) |
| Age at cohort entry, years ^a^ |  |
| <18 y | 1.34 (1.20, 1.48) |
| 18-39.9 y | 1.10 (1.07, 1.13) |
| 40-59.9 y | 1.07 (1.05, 1.10) |
| ≥60 y | 1.09 (1.07, 1.11) |
| Biopsy location ^b^ |  |
| Upper GI | 1.08 (1.07, 1.10) |
| Lower GI | 1.05 (1.03, 1.07) |
| Calendar period of cohort entry |  |
| 1969-1989 | 1.11 (1.08, 1.14) |
| 1990-1999 | 1.06 (1.04, 1.08) |
| 2000-2009 | 1.02 (1.00, 1.04) |
| 2010-2016 | 1.11 (1.07, 1.16) |
| Number of healthcare visit |  |
| 0 | 1.08 (1.06, 1.09) |
| 1 | 1.04 (1.00, 1.08) |
| 2-3 | 1.06 (1.02, 1.11) |
| ≥4 | 1.04 (0.99, 1.10) |
| ^a^ Cohort entry: date of first biopsy record for individuals with a gastrointestinal biopsy result of normal mucosa, and date of selection for the matched references | |
| ^b^ References were assigned a value of biopsy location based on the index person | |
| Conditioned on matching set (birth year, sex, county of residence, and calendar period) and further adjusted for country of birth, educational attainment, number of healthcare visits, Charlson comorbidity index, and history of GI diseases | |

| eTable 5. Risk of specific cancers during follow-up in individuals with a GI biopsy result of normal mucosa and their matched references | | | | | |
| --- | --- | --- | --- | --- | --- |
|  | Years since biopsy, HR(95%CIs) | | | | |
| Events | 1 y | 5 y | 10 y | 20 y | 30 y |
| Solid cancer | 1.18 (1.14, 1.21) | 1.00 (0.99, 1.02) | 0.99 (0.98, 1.01) | 1.05 (1.03, 1.07) | 1.08 (1.05, 1.12) |
| Any GI cancer | 1.43 (1.34, 1.53) | 0.86 (0.83, 0.90) | 0.85 (0.81, 0.88) | 0.98 (0.94, 1.03) | 1.07 (0.99, 1.15) |
| Gastric cancer | 1.36 (1.09, 1.70) | 0.90 (0.78, 1.03) | 0.92 (0.81, 1.05) | 1.16 (0.98, 1.36) | 1.31 (1.01, 1.69) |
| Colorectal cancer | 1.21 (1.11, 1.33) | 0.76 (0.72, 0.80) | 0.77 (0.73, 0.81) | 0.92 (0.86, 0.98) | 1.02 (0.92, 1.12) |
| Hepatobiliary cancer | 1.87 (1.54, 2.27) | 1.11 (0.98, 1.25) | 0.99 (0.88, 1.11) | 1.06 (0.90, 1.24) | 1.08 (0.85, 1.38) |
| Pancreatic cancer | 1.72 (1.42, 2.07) | 1.03 (0.91, 1.17) | 1.00 (0.90, 1.12) | 1.08 (0.93, 1.26) | 1.12 (0.88, 1.42) |
| Lung cancer | 1.11 (0.98, 1.26) | 0.97 (0.91, 1.04) | 0.98 (0.92, 1.05) | 1.14 (1.05, 1.24) | 1.26 (1.10, 1.44) |
| Breast cancer (women) | 1.05 (0.97, 1.15) | 1.02 (0.98, 1.07) | 1.00 (0.95, 1.04) | 1.02 (0.96, 1.07) | 1.03 (0.94, 1.12) |
| Hematologic cancer | 1.77 (1.59, 1.97) | 1.18 (1.11, 1.25) | 1.10 (1.03, 1.17) | 1.09 (1.00, 1.18) | 1.08 (0.95, 1.22) |
| Lymphoproliferative cancer | 1.64 (1.44, 1.86) | 1.15 (1.07, 1.23) | 1.08 (1.01, 1.17) | 1.06 (0.97, 1.17) | 1.04 (0.90, 1.21) |
| CIs, confidence intervals; GI, gastrointestinal; HR, hazard ratio | |  |  |  |  |
| Conditioned on matching set (birth year, sex, county of residence, and calendar period) and further adjusted for country of birth, educational attainment, number of healthcare visits, Charlson comorbidity index, and history of GI diseases | | | | | |

| eTable 6. Cumulative incidence and its difference of specific cancers during follow-up in individuals with a GI biopsy result of normal mucosa and their matched references | | | | | |
| --- | --- | --- | --- | --- | --- |
| Cumulative incidence and its difference (95% CIs), % | Years since biopsy | | | | |
|  | 1 y | 5 y | 10 y | 20 y | 30 y |
| Solid cancer |  |  |  |  |  |
| Reference | 0.30 (0.30, 0.31) | 2.83 (2.81, 2.85) | 6.30 (6.26, 6.34) | 14.55 (14.47, 14.64) | 24.40 (24.22, 24.58) |
| Normal mucosa | 0.39 (0.38, 0.41) | 3.05 (3.00, 3.10) | 6.48 (6.40, 6.55) | 14.88 (14.73, 15.02) | 25.23 (24.91, 25.55) |
| Difference | 0.09 (0.08, 0.11) | 0.22 (0.16, 0.27) | 0.17 (0.09, 0.26) | 0.32 (0.17, 0.48) | 0.82 (0.50, 1.15) |
| Any GI cancer |  |  |  |  |  |
| Reference | 0.06 (0.06, 0.06) | 0.55 (0.53, 0.56) | 1.23 (1.21, 1.25) | 3.19 (3.14, 3.24) | 6.21 (6.08, 6.35) |
| Normal mucosa | 0.11 (0.10, 0.12) | 0.61 (0.58, 0.63) | 1.18 (1.14, 1.22) | 3.01 (2.93, 3.09) | 6.14 (5.90, 6.38) |
| Difference | 0.05 (0.04, 0.06) | 0.06 (0.04, 0.09) | -0.05 (-0.09, -0.01) | -0.18 (-0.27, -0.10) | -0.08 (-0.32, 0.16) |
| Gastric cancer |  |  |  |  |  |
| Reference | 0.01 (0.00, 0.01) | 0.05 (0.05, 0.06) | 0.11 (0.11, 0.12) | 0.26 (0.25, 0.28) | 0.45 (0.42, 0.49) |
| Normal mucosa | 0.01 (0.01, 0.01) | 0.06 (0.05, 0.07) | 0.11 (0.10, 0.12) | 0.27 (0.25, 0.30) | 0.50 (0.44, 0.57) |
| Difference | 0.00 (0.00, 0.01) | 0.01 (-0.00, 0.01) | -0.00 (-0.01, 0.01) | 0.01 (-0.02, 0.03) | 0.05 (-0.01, 0.12) |
| Colorectal cancer |  |  |  |  |  |
| Reference | 0.04 (0.04, 0.04) | 0.35 (0.34, 0.36) | 0.80 (0.78, 0.81) | 2.14 (2.10, 2.19) | 4.45 (4.32, 4.58) |
| Normal mucosa | 0.06 (0.06, 0.07) | 0.33 (0.32, 0.35) | 0.67 (0.64, 0.70) | 1.83 (1.76, 1.90) | 4.10 (3.88, 4.33) |
| Difference | 0.02 (0.02, 0.03) | -0.02 (-0.03, 0.00) | -0.13 (-0.16, -0.10) | -0.31 (-0.38, -0.24) | -0.35 (-0.58, -0.12) |
| Hepatobiliary cancer |  |  |  |  |  |
| Reference | 0.01 (0.00, 0.01) | 0.05 (0.05, 0.06) | 0.13 (0.12, 0.14) | 0.33 (0.32, 0.35) | 0.63 (0.58, 0.68) |
| Normal mucosa | 0.01 (0.01, 0.01) | 0.08 (0.07, 0.09) | 0.15 (0.14, 0.17) | 0.36 (0.33, 0.39) | 0.68 (0.60, 0.77) |
| Difference | 0.01 (0.00, 0.01) | 0.02 (0.01, 0.03) | 0.02 (0.01, 0.04) | 0.03 (-0.00, 0.06) | 0.05 (-0.04, 0.13) |
| Pancreatic cancer |  |  |  |  |  |
| Reference | 0.01 (0.01, 0.01) | 0.06 (0.05, 0.06) | 0.13 (0.12, 0.14) | 0.34 (0.33, 0.36) | 0.67 (0.62, 0.72) |
| Normal mucosa | 0.02 (0.01, 0.02) | 0.08 (0.07, 0.09) | 0.15 (0.14, 0.16) | 0.37 (0.34, 0.40) | 0.73 (0.65, 0.83) |
| Difference | 0.01 (0.01, 0.01) | 0.02 (0.01, 0.03) | 0.02 (0.01, 0.03) | 0.03 (-0.00, 0.06) | 0.06 (-0.03, 0.16) |
| Lung cancer |  |  |  |  |  |
| Reference | 0.02 (0.02, 0.02) | 0.17 (0.17, 0.18) | 0.40 (0.39, 0.41) | 1.09 (1.06, 1.12) | 2.20 (2.11, 2.29) |
| Normal mucosa | 0.02 (0.02, 0.03) | 0.18 (0.17, 0.19) | 0.40 (0.38, 0.42) | 1.13 (1.08, 1.19) | 2.47 (2.30, 2.65) |
| Difference | 0.00 (-0.00, 0.01) | 0.01 (-0.01, 0.02) | -0.00 (-0.03, 0.02) | 0.05 (-0.01, 0.10) | 0.27 (0.10, 0.44) |
| Breast cancer (women) |  |  |  |  |  |
| Reference | 0.04 (0.04, 0.04) | 0.40 (0.39, 0.41) | 0.92 (0.90, 0.93) | 2.23 (2.20, 2.27) | 4.03 (3.94, 4.12) |
| Normal mucosa | 0.04 (0.04, 0.05) | 0.42 (0.40, 0.44) | 0.94 (0.91, 0.97) | 2.26 (2.20, 2.33) | 4.10 (3.94, 4.27) |
| Difference | 0.00 (-0.01, 0.01) | 0.02 (-0.00, 0.04) | 0.02 (-0.01, 0.05) | 0.03 (-0.04, 0.10) | 0.07 (-0.09, 0.24) |
| Hematologic cancer |  |  |  |  |  |
| Reference | 0.02 (0.02, 0.02) | 0.18 (0.18, 0.19) | 0.42 (0.41, 0.43) | 1.12 (1.09, 1.15) | 2.20 (2.11, 2.28) |
| Normal mucosa | 0.04 (0.04, 0.05) | 0.26 (0.25, 0.28) | 0.53 (0.51, 0.56) | 1.28 (1.23, 1.34) | 2.44 (2.29, 2.60) |
| Difference | 0.02 (0.02, 0.03) | 0.08 (0.06, 0.09) | 0.11 (0.08, 0.13) | 0.17 (0.11, 0.22) | 0.25 (0.09, 0.40) |
| Lymphoproliferative cancer |  |  |  |  |  |
| Reference | 0.01 (0.01, 0.02) | 0.14 (0.13, 0.14) | 0.32 (0.31, 0.33) | 0.83 (0.80, 0.85) | 1.62 (1.55, 1.69) |
| Normal mucosa | 0.03 (0.03, 0.03) | 0.19 (0.17, 0.20) | 0.38 (0.36, 0.41) | 0.93 (0.88, 0.98) | 1.76 (1.63, 1.90) |
| Difference | 0.02 (0.01, 0.02) | 0.05 (0.04, 0.06) | 0.07 (0.04, 0.09) | 0.10 (0.05, 0.15) | 0.14 (0.01, 0.27) |
| CIs, confidence intervals, GI, gastrointestinal | |  |  |  |  |
| Conditioned on matching set (birth year, sex, county of residence, and calendar period) and further adjusted for country of birth, educational attainment, number of healthcare visits, Charlson comorbidity index, and history of GI diseases | | | | | |

| eTable 7. Sensitivity analyses for the risk of any cancer during follow-up in individuals with a GI biopsy result of normal mucosa and their matched references | | | | | |
| --- | --- | --- | --- | --- | --- |
| Population | Years since biopsy, HR(95%CIs) | | | | |
|  | 1 y | 5 y | 10 y | 20 y | 30 y |
| With a Charlson comorbidity index of zero | 1.20 (1.16, 1.24) | 1.02 (1.00, 1.03) | 1.00 (0.98, 1.02) | 1.05 (1.03, 1.07) | 1.09 (1.05, 1.12) |
|  |  |  |  |  |  |
| Without GI diseases | 1.21 (1.17, 1.26) | 1.04 (1.02, 1.06) | 1.03 (1.01, 1.05) | 1.06 (1.03, 1.08) | 1.07 (1.04, 1.12) |
| Without endoscopy | 1.19 (1.15, 1.23) | 1.03 (1.01, 1.04) | 1.02 (1.00, 1.03) | 1.05 (1.03, 1.07) | 1.07 (1.04, 1.11) |
| Without colectomy or proctocolectomy | 1.21 (1.18, 1.25) | 1.01 (1.00, 1.03) | 1.00 (0.98, 1.02) | 1.05 (1.03, 1.07) | 1.08 (1.05, 1.12) |
| CIs, confidence intervals; GI, gastrointestinal; HR, hazard ratio | |  |  |  |  |
| Conditioned on matching set (birth year, sex, county of residence, and calendar period) and further adjusted for country of birth, educational attainment, number of healthcare visits, Charlson comorbidity index, and history of GI diseases | | | | | |

| eTable 8. Characteristics of individuals with a gastrointestinal (GI) biopsy result of normal mucosa and their siblings, a nationwide matched cohort study in Sweden, 1965-2016 | | |
| --- | --- | --- |
| Characteristics | No. (%) | |
|  | Normal mucosa (n=246,902) | Siblings (n=441,534) |
| Age at cohort entry, years ^a^ |  |  |
| Mean ± SD | 36.7 ± 16.8 | 37.7 ± 17.0 |
| Median (IQR) | 35.4 (23.5-49.5) | 37.4 (24.4-50.8) |
| <18 y | 27847 (11.3) | 57614 (13.1) |
| 18-39.9 y | 115501 (46.8) | 184699 (41.8) |
| 40-59.9 y | 78843 (31.9) | 151636 (34.3) |
| ≥60 y | 24711 (10.0) | 47585 (10.8) |
| Sex |  |  |
| Male | 96741 (39.2) | 230048 (52.1) |
| Female | 150161 (60.8) | 211486 (47.9) |
| Country of birth |  |  |
| Nordic country | 240570 (97.4) | 426297 (96.6) |
| Other country | 6332 (2.6) | 15237 (3.5) |
| Biopsy location ^b^ |  |  |
| Upper GI | 147463 (59.7) | 265416 (60.1) |
| Lower GI | 99439 (40.3) | 176118 (39.9) |
| Calendar period of cohort entry |  |  |
| 1969-1989 | 17696 (7.2) | 36248 (8.2) |
| 1990-1999 | 59485 (24.1) | 111783 (25.3) |
| 2000-2009 | 97776 (39.6) | 172040 (39.0) |
| 2010-2016 | 71945 (29.1) | 121463 (27.5) |
| Educational attainment |  |  |
| 0-9 y | 42719 (17.3) | 86133 (19.5) |
| 10-12 y | 99901 (40.5) | 173512 (39.3) |
| ≥13 y | 66150 (26.8) | 101681 (23.0) |
| Missing | 38132 (15.4) | 80208 (18.2) |
| Number of healthcare visit |  |  |
| 0 | 157394 (63.8) | 327169 (74.1) |
| 1 | 37940 (15.4) | 55151 (12.5) |
| 2-3 | 28437 (11.5) | 35548 (8.1) |
| ≥4 | 23131 (9.4) | 23666 (5.4) |
| Charlson comorbidity index |  |  |
| 0 | 208586 (84.5) | 394631 (89.4) |
| 1 | 31267 (12.7) | 39540 (9.0) |
| ≥2 | 7049 (2.9) | 7363 (1.7) |
| History before start of follow-up |  |  |
| GI disease | 113199 (45.9) | 86897 (19.7) |
| Endoscopy | 79848 (32.3) | 20629 (4.7) |
| Colectomy or proctocolectomy | 163 (0.1) | 526 (0.1) |
| Follow-up years since biopsy |  |  |
| Median (IQR) | 11.6 (6.2-18.6) | 12.1 (6.6-19.2) |
| 0.5-1 y | 2007 (0.8) | 2819 (0.6) |
| 1-4.9 y | 44947 (18.2) | 74532 (16.9) |
| 5-9.9 y | 59983 (24.3) | 103926 (23.5) |
| 10-19.9 y | 89418 (36.2) | 160523 (36.4) |
| 20-29.9 y | 41733 (16.9) | 81111 (18.4) |
| ≥30 y | 8814 (3.6) | 18623 (4.2) |
| No. of event |  |  |
| Any cancer | 18937 (7.7) | 33129 (7.5) |
| Solid cancer | 17779 (7.2) | 31162 (7.1) |
| Any GI cancer | 2316 (0.9) | 4842 (1.1) |
| Gastric cancer | 187 (0.1) | 398 (0.1) |
| Colorectal cancer | 1315 (0.5) | 2974 (0.7) |
| Hepatobiliary cancer | 276 (0.1) | 468 (0.1) |
| Pancreatic cancer | 302 (0.1) | 610 (0.1) |
| Lung cancer | 851 (0.3) | 1756 (0.4) |
| Breast cancer (women) | 1832 (2.0) | 3412 (2.1) |
| Hematologic cancer | 1158 (0.5) | 1967 (0.5) |
| Lymphoproliferative cancer | 835 (0.3) | 1505 (0.3) |
| IQR, interquartile range; SD, standard deviation | | |
| ^a^ Cohort entry: date of first biopsy record for individuals with a gastrointestinal biopsy result of normal mucosa, and date of selection for their siblings | | |
| ^b^ Siblings were assigned a value of biopsy location based on the exposed sibling | | |

| eTable 9. Association between a GI biopsy result of normal mucosa and risk of any cancer, starting follow-up from 6 months vs. 1 year after biopsy | | | | | | |
| --- | --- | --- | --- | --- | --- | --- |
| Analysis |  | Years since biopsy, HR(95%CIs) | | | | |
|  | Average HR (95%CI) | 1 y | 5 y | 10 y | 20 y | 30 y |
| Compared with matched references ^a^ |  |  |  |  |  |  |
| Starting follow-up from 6 months after biopsy | 1.07 (1.06, 1.09) | 1.21 (1.18, 1.25) | 1.01 (1.00, 1.03) | 1.00 (0.98, 1.02) | 1.05 (1.03, 1.07) | 1.08 (1.05, 1.12) |
| Starting follow-up from 1 year after biopsy | 1.04 (1.03, 1.05) | NA | 1.01 (1.00, 1.03) | 1.01 (0.99, 1.02) | 1.05 (1.03, 1.07) | 1.07 (1.04, 1.11) |
| Compared with their siblings ^b^ |  |  |  |  |  |  |
| Starting follow-up from 6 months after biopsy | 1.15 (1.13, 1.17) | 1.40 (1.33, 1.48) | 1.12 (1.09, 1.15) | 1.05 (1.02, 1.08) | 1.03 (1.00, 1.07) | 1.03 (0.97, 1.08) |
| Starting follow-up from 1 year after biopsy | 1.12 (1.10, 1.15) | NA | 1.13 (1.10, 1.16) | 1.05 (1.02, 1.08) | 1.03 (1.00, 1.07) | 1.03 (0.98, 1.08) |
| CIs, confidence intervals; GI, gastrointestinal; HR, hazard ratio; NA, not applicable | | | | | | |
| ^a^ Conditioned on matching set (birth year, sex, county of residence, and calendar period) and further adjusted for country of birth, educational attainment, number of healthcare visits, Charlson comorbidity index, and history of GI diseases | | | | | | |
| ^b^ Conditioned on family identifiers and further adjusted for birth year, sex, county of residence, calendar period, country of birth, educational attainment, number of healthcare visits, Charlson comorbidity index, and history of GI diseases | | | | | | |


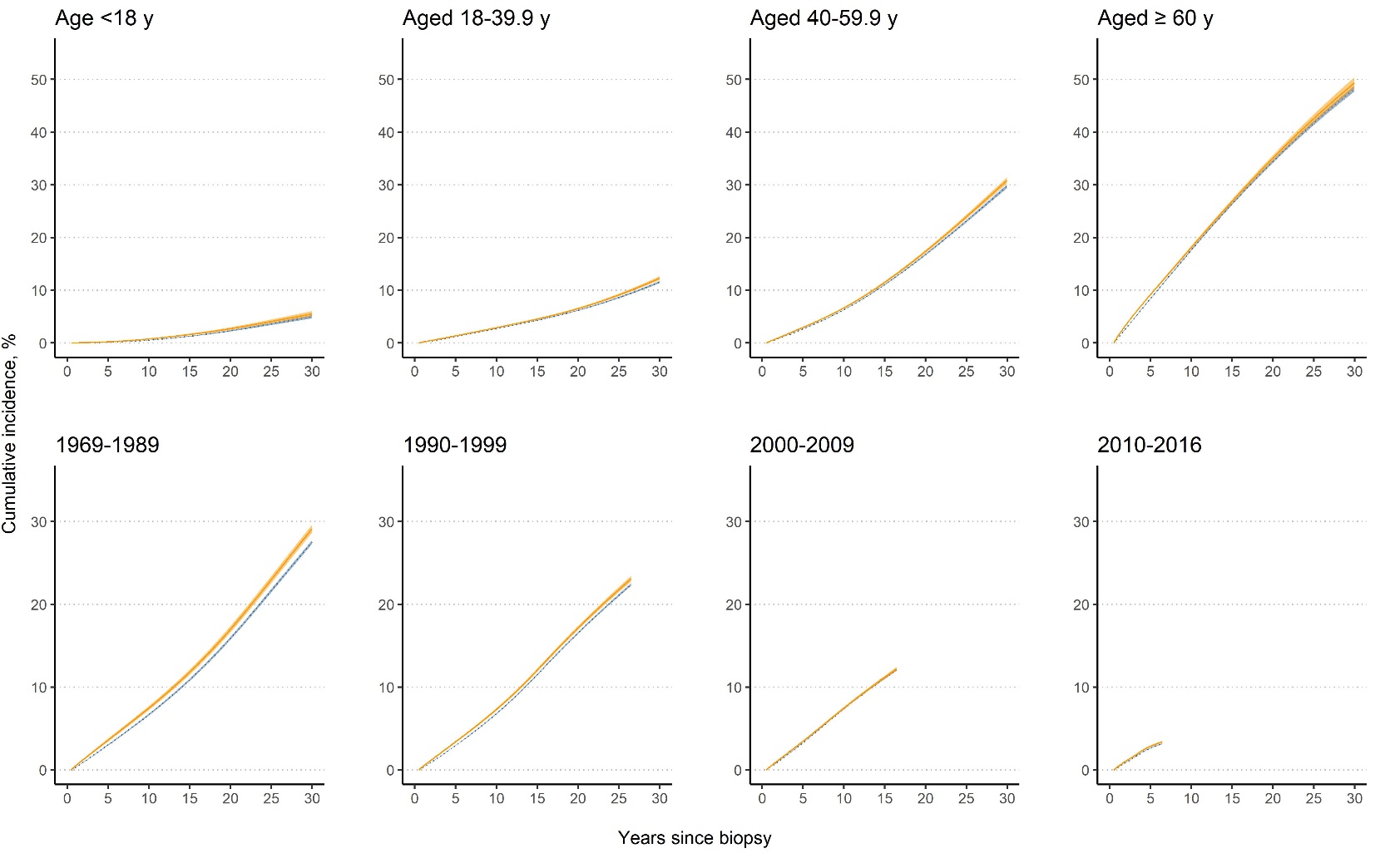


eFigure 1. Standardized cumulative incidence and 95% confidence intervals of any cancer in individuals with a GI biopsy result of normal mucosa (solid line and orange) and their matched references (dotted line and blue), stratified by age at cohort entry or calendar period of cohort entry. Follow-up was started 6 months after the biopsy.


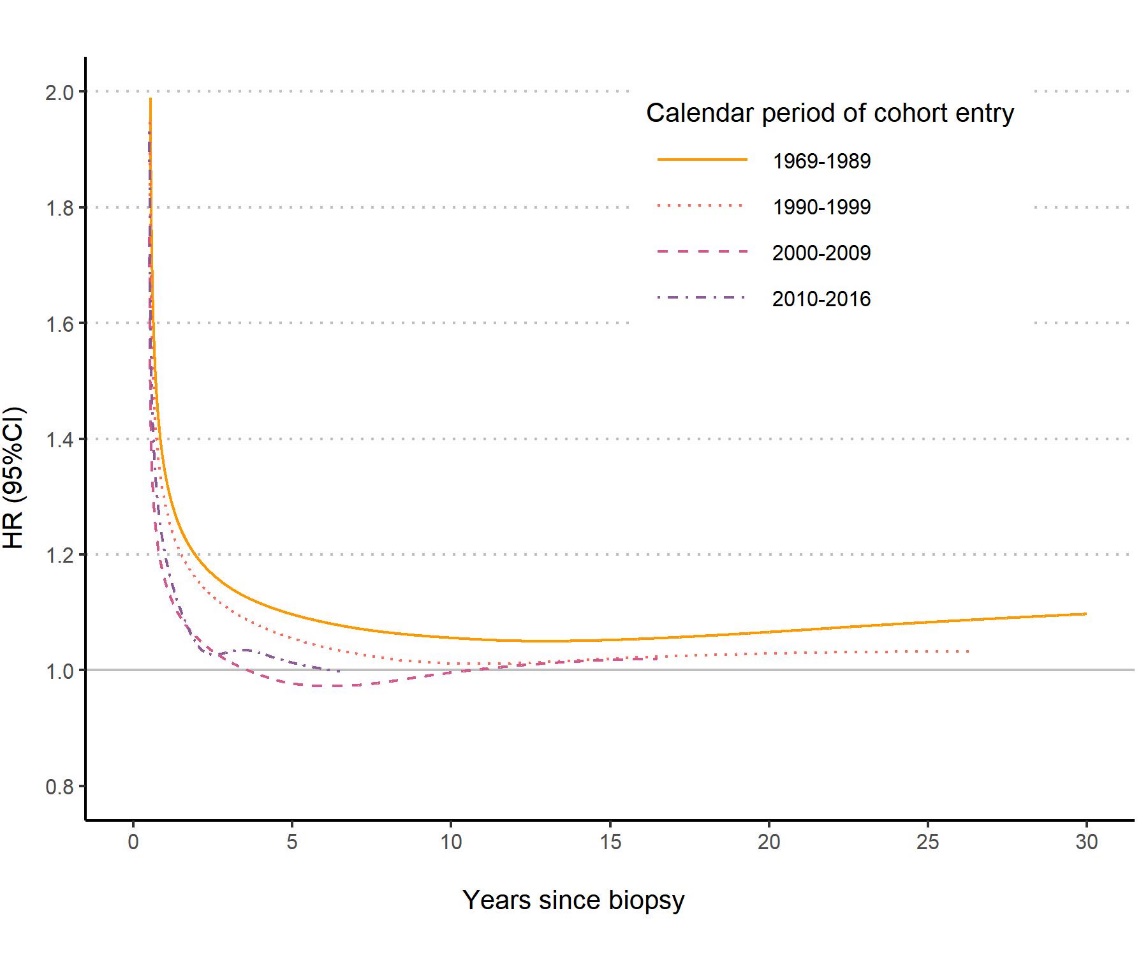


eFigure 2. Hazard ratio (HR) and 95% confidence intervals (CIs) of any cancer as a function of time since biopsy, comparing individuals with a GI biopsy result of normal mucosa with their matched references, stratified by calendar period of cohort entry.


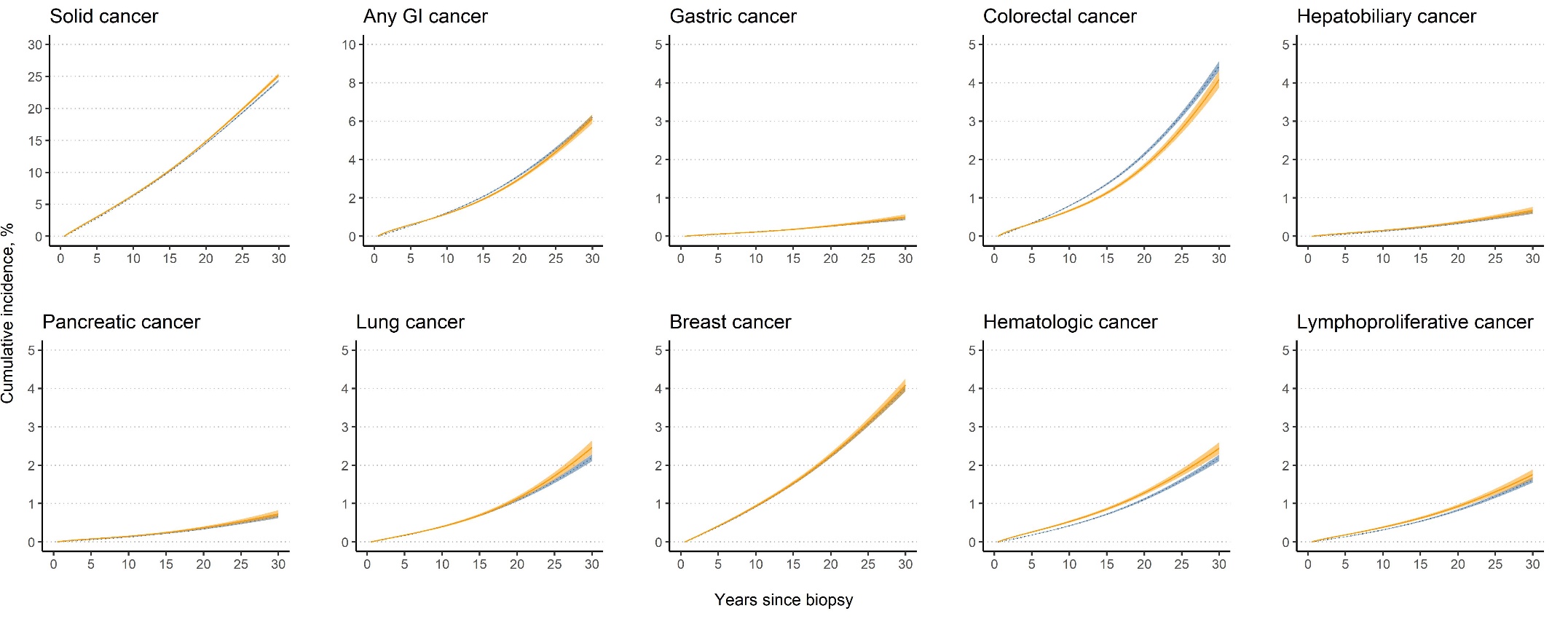


eFigure 3. Standardized cumulative incidence and 95% confidence intervals of specific cancers in individuals with a GI biopsy result of normal mucosa (solid line and orange) and their matched references (dotted line and blue). Follow-up was started 6 months after the biopsy.


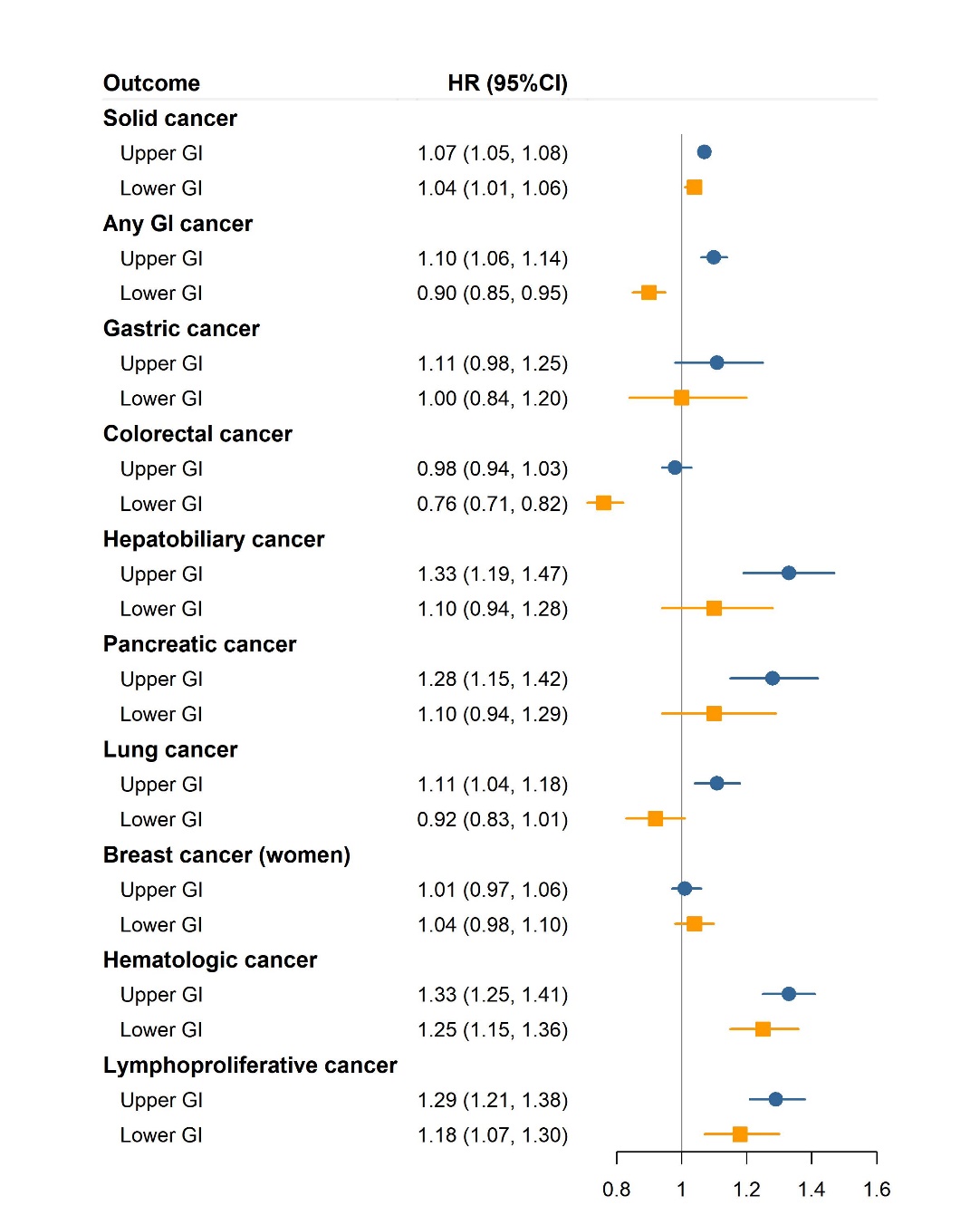


eFigure 4. Average hazard ratio (HR) and 95% confidence intervals (CIs) of specific cancers, comparing individuals with a GI biopsy result of normal mucosa with their matched references, stratified by biopsy location: upper (blue) or lower (orange) GI. Follow-up was started 6 months after the biopsy.


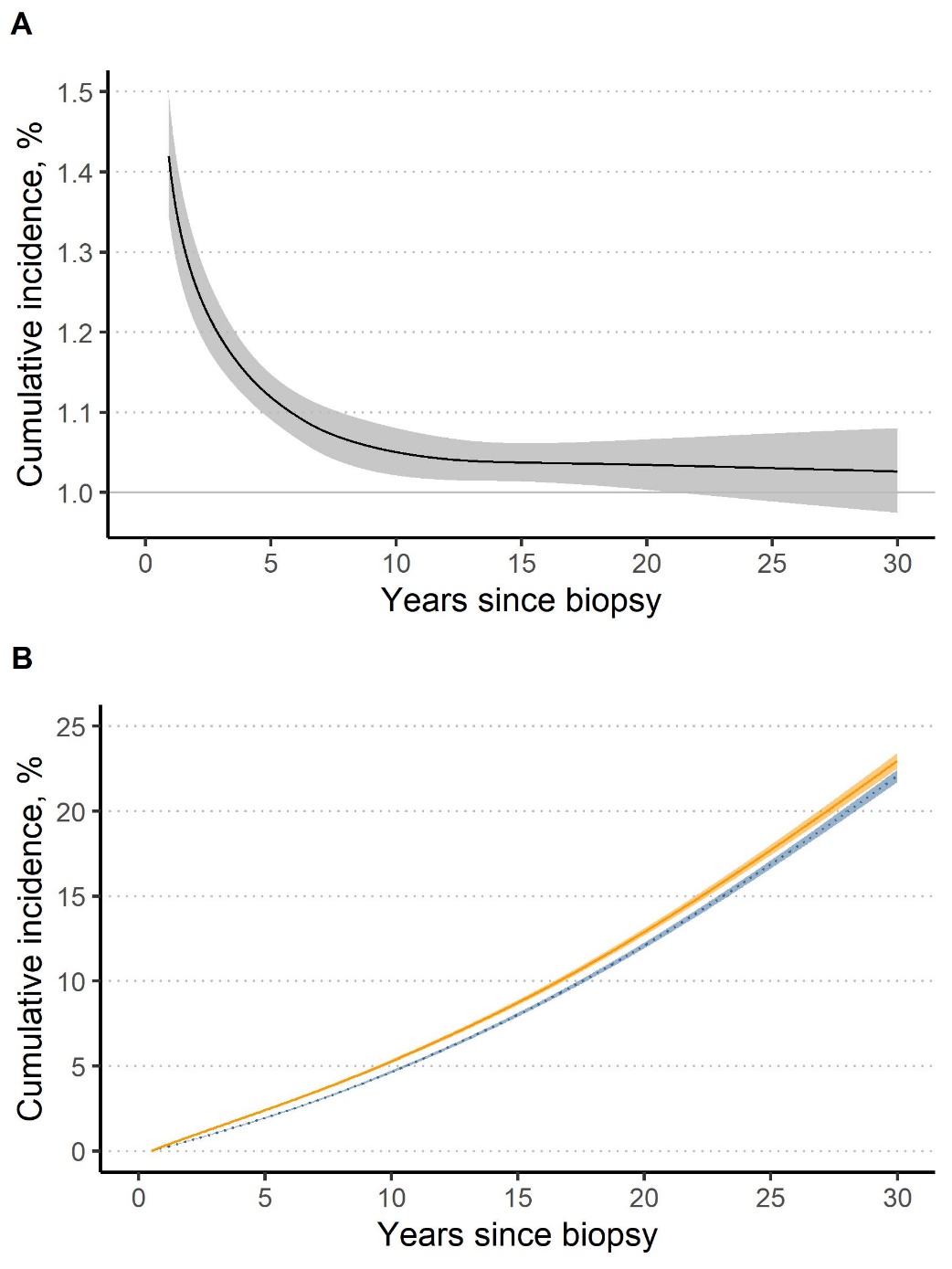


eFigure 5. (A). Hazard ratio (HR) and 95% confidence intervals (CIs) of any cancer, comparing individuals with a GI biopsy result of normal mucosa with their siblings; (B). Standardized cumulative incidence and 95% confidence intervals of any cancer in individuals with normal mucosa (solid line and orange) and their siblings (dotted line and blue). Both were estimated from the flexible parametric model and follow-up was started 6 months after the biopsy.
